# PsERF1B-PsMYB10.1-PsbHLH3 module enhances anthocyanin biosynthesis in the flesh-reddening of amber-fleshed plum (cv. Friar) fruit in response to cold storage

**DOI:** 10.1093/hr/uhad091

**Published:** 2023-05-04

**Authors:** Ranran Xu, Yubei Wang, Limin Wang, Zhilei Zhao, Jiankang Cao, Daqi Fu, Weibo Jiang

**Affiliations:** College of Food Science and Nutritional Engineering, China Agricultural University, Beijing 100083, China; College of Food Science and Nutritional Engineering, China Agricultural University, Beijing 100083, China; School of Chemical Engineering & Food Science, Zhengzhou University of Technology, Zhengzhou 450044, China; College of Quality and Technical Supervision, Hebei University, Baoding 071002, China; College of Food Science and Nutritional Engineering, China Agricultural University, Beijing 100083, China; College of Food Science and Nutritional Engineering, China Agricultural University, Beijing 100083, China; College of Food Science and Nutritional Engineering, China Agricultural University, Beijing 100083, China

## Abstract

Flesh-reddening usually occurs in the amber-fleshed plum (*Prunus salicina* Lindl.) fruit during cold storage but not during ambient storage direct after harvest. It is not clear how postharvest cold signal is mediated to regulate the anthocyanin biosynthesis in the forming of flesh-reddening yet. In this study, anthocyanins dramatically accumulated and ethylene produced in the ‘Friar’ plums during cold storage, in comparison with plums directly stored at ambient temperature. Expression of genes associated with anthocyanin biosynthesis, as well as transcription factors of *PsMYB10.1*, *PsbHLH3*, and *PsERF1B* were strongly stimulated to upregulated in the plums in the period of cold storage. Suppression of ethylene act with 1-methylcyclopropene greatly suppressed flesh-reddening and downregulated the expression of these genes. Transient overexpression and virus-induced gene silencing assays in plum flesh indicated that *PsMYB10.1* encodes a positive regulator of anthocyanin accumulation. The transient overexpression of *PsERF1B*, coupled with *PsMYB10.1* and *PsbHLH3*, could further prompt the anthocyanin biosynthesis in a tobacco leaf system. Results from yeast two-hybrid and luciferase complementation assays verified that PsERF1B directly interacted with PsMYB10.1. PsERF1B and PsMYB10.1 enhanced the activity of the promoter of *PsUFGT* individually, and the enhancement was prompted by the co-action of PsERF1B and PsMYB10.1. Overall, the stimulation of the PsERF1B-PsMYB10.1-PsbHLH3 module mediated cold signal in the transcriptomic supervision of the anthocyanin biosynthesis in the ‘Friar’ plums. The results thereby revealed the underlying mechanism of the postharvest alteration of the flesh phenotype of ‘Friar’ plums subjected to low temperature.

## Introduction

Plum (*Prunus salicina* Lindl.) is one of the mostly cultivated drupe fruit crops worldwide and more than a thousand plum cultivars are developed currently [1]. Among them, some red-, purple-, or black-skinned plum cultivars are economically significant due to their health benefits and attractive taste, such as ‘Blackamber’ [2], ‘Aozhou14’ [3], and ‘Friar’ [4]. Flesh of these plums gradually turns red from amber during cold storage and the subsequent shelf-life period, whereas the flesh remains amber in the harvested plums directly stored at room temperature. The pattern of flesh reddening (i. e. flesh blooding) is mainly impacted by storage temperature and period, as it has been demonstrated that delayed flesh reddening happens at 0 to 2°C while rapid reddening appears at 5 to 15°C [4–6]. The blooding of plum flesh is sped up when the fruit were taken out to shelf-life from cold storage, accompanied by an ethylene burst [6–8]. Cyanidin 3-*O*-glucoside has been isolated and identified as the predominant red pigment anthocyanins contributing to the cold-induced plum flesh-reddening [8–10]. Anthocyanin accumulation in plums during storage resulted from the cold-elicited activities of a serial of enzymes, such as phenylalanine ammonialyase (PAL), chalcone synthase (CHS), dihydroflavonol 4-reductase (DFR), anthocyanidin synthase (ANS), and UDP-glucose: anthocyanidin-3-*O*-glucosyltransferase (UFGT) associated with the phenylpropanoids pathway metabolism. Anthocyanins take charge of the pigmentation of tissues and organs as well as helping defense biotic and abiotic stimuli, such as plant pathogens, light and low temperature [11]. Moreover, anthocyanins are thought to have antioxidant and protective functions providing potential health-benefits for human beings [7]. Reddening flesh would be a good source for supplying food anthocyanins, because it is easily available in some plum cultivars by the transformation of amber flesh by postharvest cold stress, instead of breeding approach. Nevertheless, no report is available on how cold signal is mediated to regulate the anthocyanin production in blood-fleshed plum fruit. Even a putative element that can respond to cold signal and trigger gene expression associated with reddening is lacking, so far.

Previous studies suggested that low temperature triggers transcriptomic alterations of MYBs and MYC-like basic helix–loop–helix (bHLH) transcription factors (TFs) in plums suffering cold stress according to RNA-seq analysis [4]. It has been generally recognized that the expression of flavonoid structural genes involved in the phenylpropanoids pathway coordinately regulated by TFs, especially the ternary MYB-bHLH-WD40 (MBW) protein complex, consisting of DNA-binding R2R3-MYB TFs, bHLH TFs, and WD40-repeat proteins [12]. Different R2R3-MYBs are able to independently guide the biosynthesis of the byproducts of various flavonoid pathway branches [13]. For instance, the anthocyanin biosynthesis can be positively regulated by the homologs of *Arabidopsis* R2R3-MYBs, apple *MdMYBA*, *MdMYB1*, *MdMYB10*, and pear *PcMYB10* [14]. A series of *in vitro* and *in vivo* analyses demonstrated that MYB10 acts as a key TF regulating upstream genes to supervise the anthocyanin accumulation in sweet cherry [15, 16], pear [17], apple [14, 18], and plum fruits [19, 20]. Expression of *PsMYB10* correlates with ethylene-regulated anthocyanin biosynthesis in the plum peel [7]. PsMYB10.1 involves the promotion of the anthocyanin biosynthesis in postharvest ‘Akihime’ plum peel under light and appropriate temperature [20]. Transient overexpression of PsMYB10.2 leads to the flesh reddening in ‘Sanyueli’ during plum ripening [19]. Our previous studies implied that PsMYB10.1 may be involved in the regulation of anthocyanin biosynthesis. Therefore, whether PsMYB10.1 can regulate anthocyanin and the mechanisms of regulation has been investigated.

MYB-mediated anthocyanin biosynthetic pathway of fruit can be affected by a variety of elements, including light, low temperature, and hormone signal transduction pathways [21]. Earlier studies showed that relevant TFs and structural genes associated with the anthocyanin biosynthesis are activated to express in plants in response to low temperature [22, 23]. Cold-activated MBW complexes are created through the up-regulation of critical positive genes of anthocyanin biosynthesis [21]. In addition, various TFs can interact with the MBW complex in the regulation of anthocyanin biosynthesis. CBFs (C-repeat binding factors) physically interact with SmMYB113 and promote the activation of *SmCHS* and *SmDFR*, which facilitate the anthocyanin accumulation under cold conditions in Arabidopsis [23]. MdERF38, an ethylene response factor (ERF), binds to *MdMYB1* promoter to stimulate anthocyanin production respond to drought stress [24]. Arabidopsis AtERF4 and AtERF8 participate in light-modulated anthocyanin biosynthesis [25]. Pp4ERF24 and Pp12ERF96 regulate blue light-induced anthocyanin biosynthesis via binding with PpMYB114 and encouraging the interaction between PpMYB114 and PpbHLH3 [26]. PyERF3 collaborates with PyMYB114 and PybHLH3 to co-modulate the biosynthesis of anthocyanins in pear fruit [27]. ERFs may correlate with ethylene-regulated anthocyanin biosynthesis in the flesh of plums [7]. In our previous work, we found that the expression of *PsbHLH3* was significantly up-regulated in flesh-reddening in ‘Friar’ plums stored at 0°C 4 and 6 w of cold storage [8]. Meanwhile, *PsERF1B* was activated during cold storage and the following shelf life [8]. Therefore, whether PsbHLH3 and PsERF1B participate in the biosynthesis of anthocyanins has been investigated.

Amber-fleshed plum (*P. salicina* Lindl. cv Friar) fruit often suffers flesh-reddening during cold storage [3, 6, 8]. The phenotype alteration is of importance for obtaining different coloration of inner flesh of fruit by means of postharvest regulation with cold stress, instead of long-term breeding. Accordingly, the study was aimed to investigate the transcriptional mechanism of the anthocyanin metabolism in the blood-fleshed plums during cold storage. The results from RNA-seq suggested that PsMYB10.1 and PsERF1B might be involved in the response to cold and the regulation of anthocyanin biosynthesis. The function of PsMYB10.1 on the supervision of the anthocyanin biosynthesis in flesh reddening of ‘Friar’ plum was to be validated. The interaction between PsERF1B and PsMYB10.1 on prompting the anthocyanin biosynthesis was to be further studied. The results would imply an PsERF1B–PsMYB10.1 protein complex in the postharvest regulation of cold-stimulated anthocyanins accumulation in fruit flesh.

## Results

### Storage at low temperature stimulated flesh-blooding of ‘friar’ plum fruit

**Figure 1:**
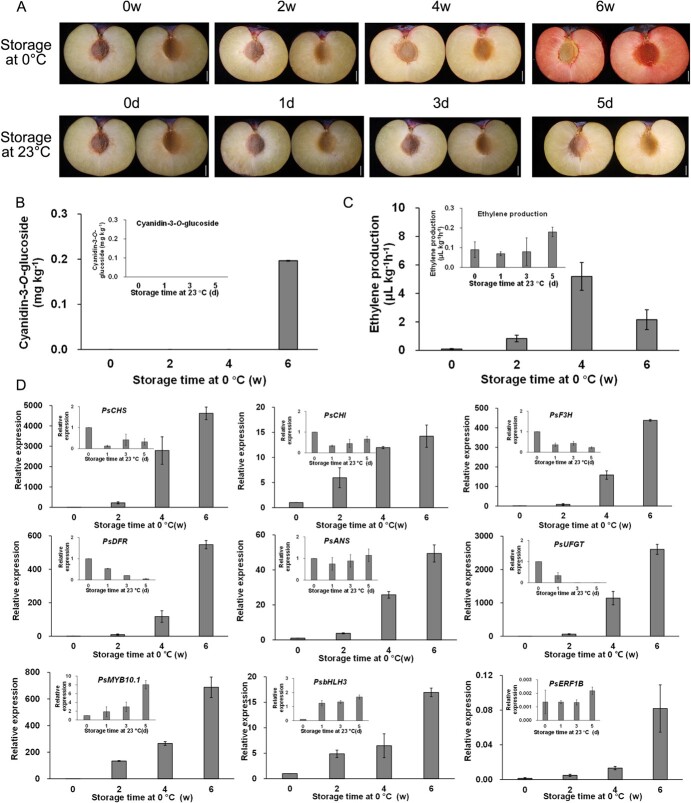
Changes of flesh phenotype (**A**), the content of cyanidin-3-*O*-glucoside (**B**), ethylene production (**C**), and gene expression (**D**) in ‘Friar’ plum fruit during cold and ambient storage. Each value is the mean for three replicates. The vertical bar indicates the standard error. Scale bar = 1 cm.

The flesh of ‘Friar’ plums remained amber-yellow during ripening at ambient temperature immediately following harvest, while the plum fruit developed flesh- blooding during cold storage at 0°C for 6 w (Fig. 1A). Correspondingly, cyanidin-3-*O*-glucoside was the only red pigment detectable and sharply accumulated in flesh-blooding during cold storage (Fig. 1B). Besides, a peak of ethylene release was detected in the fruit after 4 w of storage before flesh-reddening after 6 w of storage, whereas the release of ethylene remained at a remarkedly low level during ambient temperature, similar to that at harvest (Fig. 1C). The results suggested that ethylene might be involved in the activation of flesh reddening.

In order to understand the expression of main anthocyanin biosynthetic genes in the flesh of plums as stimulated by low temperature, qRT-PCR analysis was conducted. Results suggested that the expression levels of *PsCHS, PsCHI* (*chalcone isomerase*)*, PsF3H* (*flavanone 3-hydroxylase*)*, PsDFR*, *PsANS,* and *PsUFGT* were increased during cold storage (Fig. 1D). In particular, expressions of *PsDFR*, *PsANS*, and *PsUFGT* genes, encoding enzymes that directly catalyze the anthocyanins production, was up-regulated obviously after 4 w and increased dramatically after 6 w of storage. In contrast, these genes remained inactive and were even down-regulated in the plums stored at room temperature. In our previous work, *PsMYB10.1*, a R2R3-MYB transcription factors, was found much upregulated in the flesh of ‘Friar’ plums in response to cold storage, based on the data of log_2_foldchange (ColdS4w/Harvest) and log_2_foldchange (ColdS6w/Harvest) (Table S1, see online supplementary material). For further investigation on the transcriptional regulation of structural genes, the gene expression of *PsMYB10.1*, *PsbHLH3*, and *PsERF1B* was detected. It was found that these TFs were substantially up-regulated in reddening flesh in a great extent during cold storage (Fig. 1D).

### Ethylene signal transduction was indispensable for flesh reddening of ‘friar’ plum fruit

**Figure 2:**
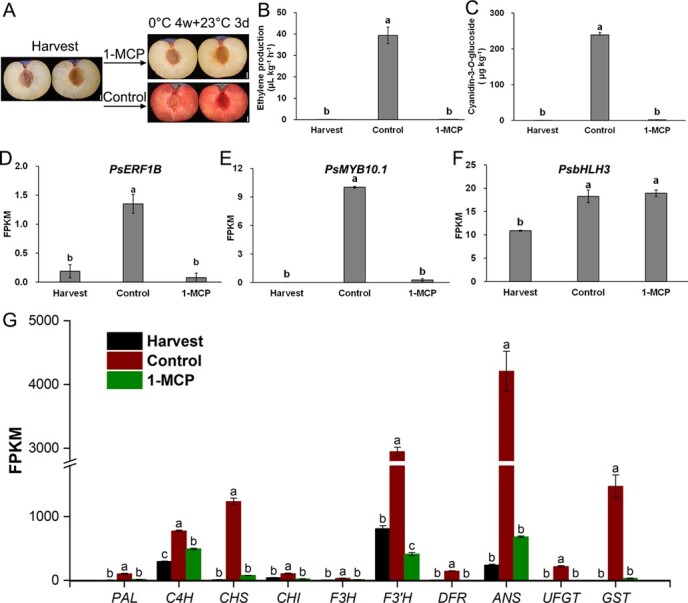
Effects of 1-methylcyclopropene (1-MCP) treatment on phenotypes (**A**), ethylene production (**B**), the content of cyanidin-3-*O*-glucoside (**C**), the expression of transcription factors (**D**, **E**, and **F**) and structural genes (**G**) in ‘Friar’ plums. Each value is the mean for three replicates. The vertical bar indicates the standard error. Values marked with different lowercase letters were significantly (*P* < 0.05) different within the same gene according to Tukey’s test. Scale bar = 1 cm.

Because almost none of ethylene release was detected in the amber-fleshed plums, and a peak ethylene release appeared after 4 w of storage prior to flesh-reddening after 6 w of storage, a shortage of ethylene might lead to the failure of flesh reddening. In order to verify the view, ‘Friar’ plums were treated with 1-methylcyclopropene (1-MCP), an inhibitor of ethylene act by competitively blocking ethylene-receptors. Unlike the control, the flesh didn’t turn red in the 1-MCP-treated plums subjected to clod storage (Fig. 2A). The production of ethylene was fully suppressed in the 1-MCP-treated plums after 3 d of shelf-life at 23°C after the end of the four-week cold storage (Fig. 2B). Cyanidin-3-*O*-glucoside, the red pigment, failed to produce in the 1-MCP-treated plums (Fig. 2C). The gene expression of *PsERF1B* and *PsMYB10.1* was almost completely inhibited by the 1-MCP treatment, though no effect on the expression of *PsbHLH3*, compared to the control (Fig. 2D, E, and F). Further investigation suggested that the expression of *PsPAL, PsC4H* (*cinnamate 4-hydroxylase*)*, PsCHS, PsCHI, PsF3H, PsF3’H* (*flavanone 3′-hydroxylase*)*, PsDFR, PsANS, PsUFGT*, and *PsGST* (*glutathione S-transferase*) associated with the anthocyanin biosynthesis were suppressed by the 1-MCP treatment, compared to the control (Fig. 2G). The results implied that the cold stress signal might be mediated via the ethylene signal transduction pathway. Blocking ethylene act might stop the mediation and therefore the cold storage failed to activate the anthocyanin biosynthesis in the plums.

### Phylogenetic analysis and multiple sequence alignment

PsMYB10.1 was aligned with MYBs in other Rosacea species and model plant Arabidopsis. Phylogenetic analysis showed that PsMYB10.1 was located in the anthocyanin clade (SG6), which mainly contains anthocyanin-activating MYB proteins (Fig. S1A, see online supplementary material). PsMYB10.1 was closely related to cherry plum PcMYB10.1. The sequence alignment of PsMYB10.1 showed that there was a highly conserved R2R3 domain, with a bHLH-binding domain, located in the N-terminus of the amino acid sequence. The sequence of ANDV and SG6, which are two distinguishing motifs of anthocyanin-MYB promoter, was found in the C-terminus of PsMYB10.1 (Fig. S1B, shown by red dotted boxes, see online supplementary material). The phylogenetic analysis presented that PsERF1B fell in the group IX and was identified with high homology with PmERF1B (Fig. S1C, see online supplementary material). An AP2 motif was found in the PsERF1B sequence according to a multiple protein sequence alignment (Fig. S1D, see online supplementary material).

### PsMYB10.1 positively regulates anthocyanin biosynthesis in plum fruit

**Figure 3:**
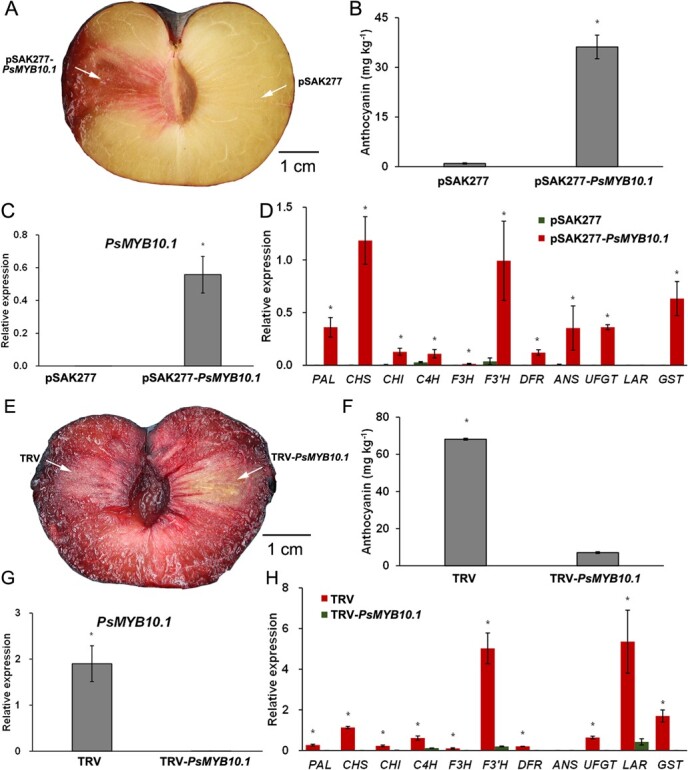
Analysis of the physiological function of PsMYB10.1 in plum fruit. **A**–**D**, transient overexpression of *PsMYB10.1*. **E**–**H**, silencing of *PsMYB10.1*. **A** and **E**, phenotype. Scale bar = 1 cm. **B** and **F**, the content of anthocyanins. **C** and **G**, the expression level of *PsMYB10.1*. **D** and **H**, the expression of structural genes related to anthocyanin biosynthesis. Each value is the mean for three replicates. The vertical bar indicates the standard error. Values marked with ‘^*^’ were significantly (*P* < 0.05) different within the same gene according to *t* test.

Transient overexpression of *PsMYB10.1* stimulated reddening in the flesh of ‘Friar’ plums that should have been amber-yellow at ambient temperature (Fig. 3A). A considerably higher anthocyanin content was detected in the reddening area with *PsMYB10.1* overexpression than that with the empty vector (Fig. 3B). The expression of *PsMYB10.1* was greatly up-regulated in the pSAK277-*PsMYB10.1* infected area, while none was expressed in the area infected with the empty vector, denoting that the gene was successfully transferred into the flesh and overexpressed (Fig. 3C). The transient overexpression of *PsMYB10.1* activated the anthocyanin biosynthetic genes of *PsPAL, PsCHS, PsCHI, PsC4H, PsF3H, PsF3’H, PsDFR, PsANS, PsUFGT,* and *PsGST* in pSAK277-*PsMYB10.1* infected flesh, in comparison with almost no expression in the empty vector infected area (Fig. 3D).

In order to further demonstrate the function of the PsMYB10.1, the virus-induced gene silencing (VIGS) system was employed to momentarily inhibit the expression of *PsMYB10.1* in the flesh of ‘Friar’ plums that were supposed to be reddening during shelf-life after storing at low temperature for 4 w. Red coloration in the infiltrated location of flesh was much alleviated in the ‘Friar’ plums after cold storage by injection of pTRV-*PsMYB10.1*, whereas dark reddening occurred in the fruit after cold storage with the only injection of pTRV (Fig. 3E). The results can be attributed to the greatly reduced the anthocyanins accumulation in the pTRV-*PsMYB10.1*-infected flesh, against the mass anthocyanins accumulation caused by cold stress in the flesh area with the injection of empty vector (Fig. 3F). Additionally, the expression level of *PsMYB10.1* was inhibited as a result of VIGS-silencing of *PsMYB10.1* (Fig. 3G). Besides, the silencing of *PsMYB10.1* almost led to a complete prevention of the expression of structural genes related to anthocyanin biosynthesis, such as *PsPAL, PsCHS, PsCHI, PsC4H, PsF3H, PsF3'H, PsDFR, PsANS, PsUFGT, PsLAR*, and *PsGST* (Fig. 3H).

### PsMYB10.1 activates the transcription of *PsUFGT* and the activation is enhanced by PsERF1B

**Figure 4:**
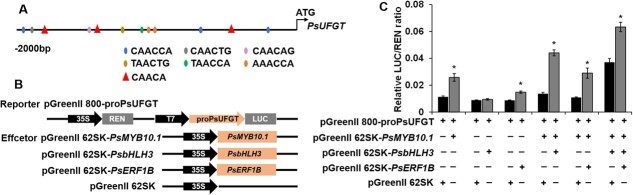
*PsMYB10.1*, *PsbHLH3*, and *PsERF1B* co-transfection activates the *PsUFGT* promoter. **A**, the prediction of the *cis*-acting elements in the promoter of *PsUFGT*, performed using the PlantCARE (http://bioinformatics.psb.ugent.be/webtools/plantcare/html/) database. Different color ellipses represent predicted MYB binding sites. Red triangles represent predicted ERF binding sites. **B**, schematic diagrams of effector and reporter vectors used for the dual-luciferase assay. **C**, the activation of PsMYB10.1, PsbHLH3, and PsERF1B on the promoter of *PsUFGT* gene using dual-luciferase reporter assay in *Nicotiana benthamiana* leaves. Each value is the mean for three replicates. The vertical bar indicates the standard error. Values marked with ‘^*^’ were significantly (*P* < 0.05) different between two groups according to *t* test.

UFGT, the final enzyme that catalyzes the glycosylation of anthocyanidins in the anthocyanin biosynthesis pathway, plays an important role in the biosynthesis of anthocyanins. Cold storage activated the high expression level of *PsUFGT*. To investigate whether PsERF1B, PsMYB10.1, and PsbHLH3 activate the expression of structural genes in the anthocyanin biosynthesis pathway, the *cis*-acting elements in the promoter of *PsUFGT* were analysed. The results showed that there were multiple potential MYB binding sites located in the promoter of *PsUFGT*, such as CAACCA, CAACTG, and CAACAG (Fig. 4A). Besides, three RAA motifs (ERF binding motifs, CAACA) were found in the promoter of *PsUFGT.* Results obtained from dual-luciferase reporter experiments in tobacco leaves showed that both PsERF1B and PsMYB10.1 could promote the activity of the promoter of *PsUFGT* (proPsUFGT), respectively, although PsbHLH3 by itself cannot influence the activity of proPsUFGT (Fig. 4C). Co-transfection of PsMYB10.1 and PsbHLH3 had a higher activity of the *PsUFGT* promoter than PsMYB10.1 alone. Co-transfection of PsMYB10.1 and PsERF1B further increased the activity of proPsUFGT, while the co-transfection of PsMYB10.1-PsbHLH3-PsERF1B stimulated the most increase in the activity of the proPsUFGT.

### PsERF1B prompts the PsMYB10.1-mediated anthocyanin biosynthesis

**Figure 5:**
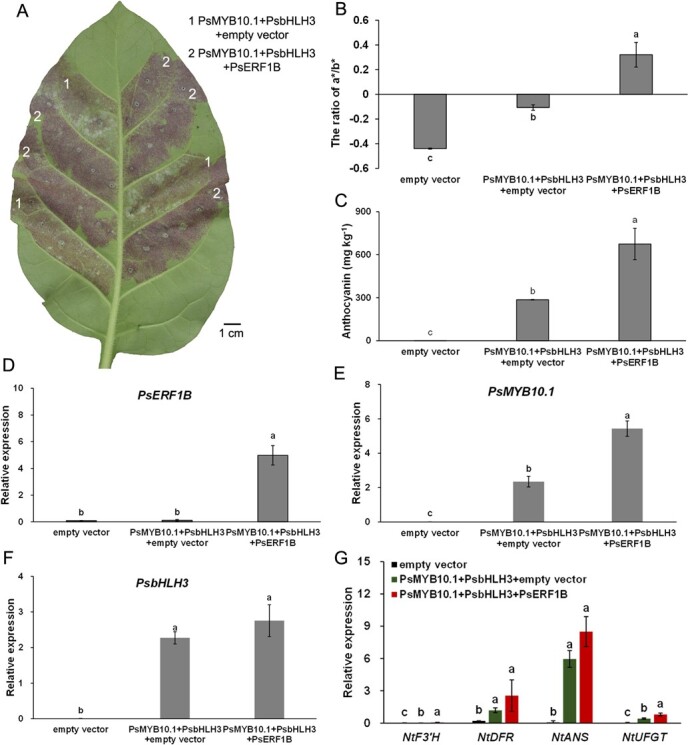
PsERF1B prompts PsMYB10.1-mediated anthocyanin biosynthesis in *Nicotiana tabacum* leaves. **A**, phenotype. Scale bar = 1 cm. **B**, the ratio of a^*^/b^*^. **C**, the content of anthocyanins. **D**, **E**, and **F**, the expression level of *PsERF1B*, *PsMYB10.1* and *PsbHLH3*. **G**, the expression level of structural genes related to anthocyanin biosynthesis. Each value is the mean for three replicates. The vertical bar indicates the standard error. Values marked with different lowercase letters were significantly (*P* < 0.05) different according to Tukey’s test.

In order to explore the function of PsERF1B in regulating anthocyanin biosynthesis, transient overexpression of *PsERF1B*, *PsMYB10.1*, and *PsbHLH3* individually or in combination in tobacco leaves were performed and verified by qRT-PCR amplification. Although neither *PsMYB10.1* nor *PsbHLH3* overexpression alone caused reddening of tobacco leaves after infiltration for 3 d, the co-transfection of *PsMYB10.1* and *PsbHLH3* together led to a faint reddening in the tobacco leaves (Fig. S2, see online supplementary material). The results suggested that PsMYB10.1 and PsbHLH3 act together in the regulation of anthocyanin biosynthesis in tobacco leaf system. Nonetheless, *PsERF1B* co-transfected with *PsMYB10.1* and *PsbHLH3* (i.e. the PsERF1B-PsMYB10.1-PsbHLH3 module), led to a more clearly dark red area in tobacco leaves than the transfection of *PsMYB10.1* and *PsbHLH3* (Fig. 5A). The ratio a*/b* indicates the degree of redness in tobacco leaves. The ratio a*/b* reached 0.32 in tobacco leaf area co-injected with *PsERF1B*-*PsMYB10.1-PsbHLH3*, which was obviously higher than the ratio measured after the only transfection of *PsMYB10.1* and *PsbHLH3* (Fig. 5B). The total anthocyanins content was greatly increased by the co-transfection of *PsERF1B* to the combination of *PsMYB10.1* and *PsbHLH3* than that without *PsERF1B* (Fig. 5C). The expression of *PsERF1B* was greatly up-regulated in the area co-injected with *PsERF1B*-*PsMYB10.1-PsbHLH3*, while none was expressed in the area infected with *PsMYB10.1* and *PsbHLH3*, denoting that the gene was successfully transferred into the flesh and overexpressed. (Fig. 5D). Furthermore, the co-transfection of *PsERF1B*-*PsMYB10.1-PsbHLH3* promoted the expression of *PsMYB10.1*, compared to the co-transformation of *PsMYB10.1* and *PsbHLH3* (Fig. 5E), though the module didn’t have an effect on the expression of PsbHLH3 (Fig. 5F), compared to the only co- transfection of *PsMYB10.1* and *PsbHLH3*. The co-transfection of *PsERF1B-PsMYB10.1-PsbHLH3* in the tobacco leaves activated the expression of *PsUFGT*, the important structural genes directly catalyzing the anthocyanin production, against the only co-transfection of *PsMYB10.1-PsbHLH3* (Fig. 5G). These findings implied that PsERF1B strengthened the expression of PsMYB10.1, which upregulates anthocyanin biosynthesis, and thereby accelerated anthocyanin accumulation, resulting in tissue reddening.

### PsERF1B interacts with PsMYB10.1

**Figure 6:**
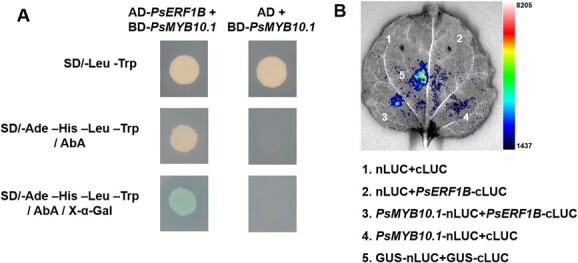
PsERF1B interacted with PsMYB10.1. **A**, Y2H assays. −Leu –Trp means SD medium lacking Leu and Trp. −Ade − His −Leu − Trp means SD medium lacking Leu, Trp, His, and Ade. AbA means SD medium containing Aureobasidin A. X-α-gal means SD medium containing X-α-gal. The empty pGADT7 vector was used as the negative control. **B**, Luciferase complementation Assay. GUS protein was used as positive control.

Yeast two-hybrid (Y2H) assay was employed to analyse the protein interaction between PsERF1B and PsMYB10.1. Firstly, the self-activation activity of PsERF1B and PsMYB10.1 proteins was tested, respectively. The result showed that only PsMYB10.1 protein had strong self-activation activity. Therefore, Aureobasidin A (AbA) was added into the SD medium (−Leu –Trp –His –Ade) to inhibit the self-activation activity of PsMYB10.1. Yeast cells harboring pGBKT7-*PsMYB10.1* and pGADT7-*PsERF1B* grew on SD/ –Leu –Trp –His –Ade media containing AbA and X-α-Gal (Fig. 6A). Conversely, yeast cells harboring pGBKT7-PsMYB10.1 and pGADT7 did not grow. These observations revealed that PsERF1B was able to interact with PsMYB10.1. To further investigate the *in vivo* interaction between PsERF1B and PsMYB10.1, luciferase complementation assay was performed. The co-expression of PsERF1B and PsMYB10.1 in the tobacco leaf resulted in a strong bioluminescence signal, indicating that PsERF1B and PsMYB10.1 interacted with each other *in vivo* (Fig. 6B).

## Discussion

It has allegedly been reported that the flesh of some red, purple or black-skinned plum cultivars progressively turns red from amber during cold storage or after removal, but remains amber during the entire storage at room temperature [3, 5, 6, 8, 11, 28–30]. Such alteration of flesh-reddening provides an attractive phenotypic trait of the fruit as a result of the anthocyanin accumulation. However, the molecular mechanism of anthocyanin biosynthesis in the stored plums as it responded to low temperature has not been thoroughly characterized. Anthocyanin biosynthesis is a multiplex progress involving the regulated management of core structural genes and generally transcriptionally guided by MBW protein complex, especially R2R3-MYB and bHLH TFs [13]. In this study, the positive regulation of PsMYB10.1 on the anthocyanin biosynthesis during flesh reddening was further confirmed. Moreover, we found that an ethylene response factor PsERF1B strengthens the expression of PsMYB10.1. There may be a formation of PsERF1B-PsMYB10.1-PsbHLH3 module that upregulates genes associated with anthocyanin biosynthesis and thereby leads to flesh-reddening of amber-fleshed ‘Friar’ plums subjected to cold storage.

It is well established that MYBs regulate anthocyanin production in response to low temperature and other environmental stress [31]. Previous reports have demonstrated that PsMYB10.1 promotes the anthocyanin biosynthesis by activating the promoters of *PsANS*, *PsUFGT*, and *PsGST* in ‘Akihime’ plum peel [20]. PsMYB10.2 can activate the expression of *PsUFGT* and *PsGST* in the anthocyanin pathway activator in the flesh of ‘Sanyueli’ plums [19]. As the final enzyme in the anthocyanin biosynthetic pathway, UFGT directly promotes the biosynthesis and stabilization of anthocyanins. The activation of the promoter of *PsUFGT* is of importance in the anthocyanin biosynthesis. In addition to multiple MYB binding sites, several RAA motifs were located in the promoter of *PsUFGT*. RAA motifs permit ERF genes bind to downstream genes [32]. In the present study, PsMYB10.1 could bind on the promoter of *PsUFGT* and therefore activate the transcription of *PsUFGT*. Furthermore, the PsERF1B-PsMYB10.1-PsbHLH3 module more strongly enhanced the activation of the promotor of *PsUFGT*. These results presented a novel insight to the underlying mechanism of the regulation of anthocyanin biosynthesis in ‘Friar’ plums suffering cold stress.

Several reports have implied that ERFs interact with multiple transcription factor proteins in plants in response to cold stress [33]. Interactions between ERFs and MYBs have also been previously reported. Previous research demonstrated that MdERF1B binds to the promoter of *MdMYB9/11* as well as directly interacts with MdMYB9/11 protein and consequently increases anthocyanin and proanthocyanidin accumulation in apples [32]. Besides, MdERF1B mediates ethylene and jasmonic acid to modulate anthocyanin production by directly upregulating the expression of *MdMYC2* and *MdMYB1/9/11* [34]. In this study, PsERF1B fell in the ERF group IX, which is closely related to TFs that are involved in the ethylene signal pathway responding to abiotic defense (Fig. S1B, see online supplementary material) [35]. Inhibition of ethylene act with 1-MCP caused the none of the expression of *PsERF1B* and lack of anthocyanin accumulation in the ‘Friar’ plums even stored at low temperature. Results from our studies suggested that cold stress signal might activate PsERF1B via ethylene signal transduction pathway, and then the PsERF1B prompted the expression of *PsMYB10.1*, which directly upregulates the anthocyanin biosynthesis. The results from Y2H and luciferase complementation assay showed that PsERF1B directly interact with PsMYB10.1. Accordingly, the collaborative relationship between PsERF1B and PsMYB10.1 in plums should be further investigated. Nevertheless, the cold-triggered change of *PsERF1B* expression was similar to previous reports on Trifoliate orange *PtrERF108* that directly activates the *PtrRafS* promoter to modify raffinose biosynthesis and increase cold tolerance [36]. MdERF1B improves the activation of the MdCBF1 promoter to enhance apple resistance to cold, with the involvement of MdCIbHLH1 [37]. In addition, a birch ERF transcription factor, BpERF13, upregulates two CBF genes and four reactive oxygen species scavenging genes respond to cold stress [38]. PsERF1B might also regulate CBF or other TF genes in plums to change fruit resistance to cold stress, though further study is still needed.

**Figure 7:**
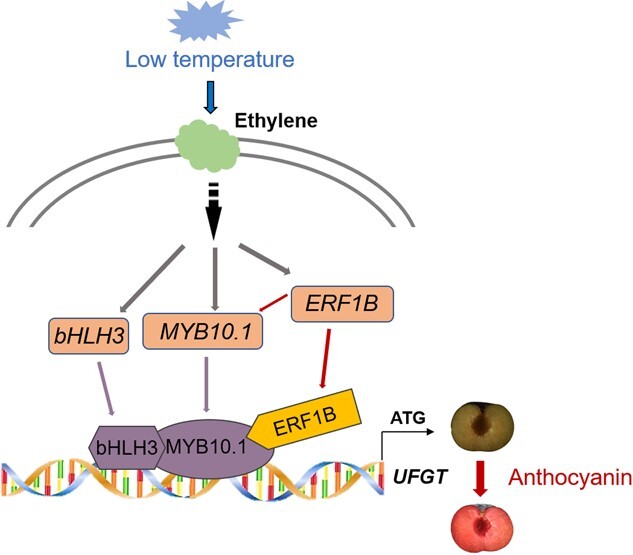
A model of PsERF1B-mediated anthocyanin accumulation in response to cold stress in the ‘Friar’ plums. The PsERF1B-PsMYB10.1-PsbHLH3 module enhances the activation of the promoter of *PsUFGT*, which promotes anthocyanin accumulation leading to fleshing reddening under low temperature.

As one of ethylene response factors, PsERF1B plays crucial role in a variety of stress responses under the guidance of ethylene [37]. In plums, an ethylene burst was found during shelf-life period after cold storage, along with the sharp pigmentation in the flesh [8]. The application of 1-MCP totally inhibited flesh reddening in plum fruit [3]. *PsERF1B, PsMYB10.1* and anthocyanin biosynthetic genes were completely silenced in response to 1-MCP treatment, accompanied by the absence of biosynthesis of anthocyanin in ‘Friar’ plums stored at low temperature. Overall, it can be hypothesized that cold signal stimulates the expression of *PsERF1B* via the ethylene transduction pathway, and PsERF1B prompts PsMYB10.1 acting on downstream genes associated with the anthocyanin biosynthesis (Fig. 7). In particular, the PsERF1B-PsMYB10.1-PsbHLH3 module more strongly enhanced the activation of the promotor of *PsUFGT*, consequently resulting in flesh reddening in the ‘Friar’ plums subjected to cold storage.

## Conclusion

In conclusion, PsERF1B mediated the low temperature stress and interacted with PsMYB10.1 in ‘Friar’ plums during storage under low temperature. PsERF1B, PsMYB10.1, and PsbHLH3 together promoted the activation of *PsUFGT* leading to the accumulation of anthocyanins and consequently flesh-reddening in the plum. The findings would offer a fundamental understanding on the cold stimulated regulation of anthocyanin biosynthesis, and provide a promising approach for postharvest modification of the properties of fruit flesh phenotype to meet different requirements by consumers.

## Materials and methods

### Plant material and processing

*P. salicina* Lindl. cv ‘Friar’ plum at the commercial mature stage were harvested from Haidian, Beijing, China. The fruits were classified into two groups. One group (approximately 600 plums, containing three replicates) were stored directly at 23°C for five days after harvest. The other group (roughly 1800 plums, containing three replicates) were stored at 0°C for six weeks. Three biological replicates were prepared for each sample, with ten fruits used for biological replicate.

For the 1-MCP treatment, harvested ‘Friar’ plums were treated with 1 μL L^−1^ 1-MCP for one day. Subsequently, the plums were stored at 0°C for four weeks and subsequently 23°C for three days. Plums treated without 1-MCP were used as the control.

### Measurements of contents of total anthocyanins, cyanidin-3-O-glucoside, and ethylene production

pH-Differential spectrophotometry was employed in the measurement of the total anthocyanins [30]. The cyanidin-3-*O*-glucoside content was measured by the use of ultra-performance liquid chromatography tandem mass spectrometer according to Xu *et al.* [8]. A gas chromatograph (Model 7890F; Tianmei Co., Shanghai, China) was used to examine ethylene production.

### RNA extraction, library construction, and sequencing

RNAs from the flesh of freshly harvested fruit (Harvest) and plums stored at 0°C for four weeks (ColdS4w) and six weeks (ColdS6w) were isolated. In addition, RNAs from the flesh of plums treated with 1-MCP and the control were isolated. The high-quality RNA extraction, sequencing library construction and sequencing were performed according to Xu *et al.* [8]. The mapped genome sequence was obtained from ‘Sanyueli’ plum (https://www.rosaceae.org/Analysis/9450778) [39].

### Phylogenetic analysis and multiple sequence alignment

The deduced amino acid sequences of PsMYB10.1 and other MYBs, PsERF1B and other ERFs were aligned by DNAMAN (version 6, Lynnon Biosoft, San Ramon, CA, USA). The MEGA software (v6.0) [40] was employed for the phylogenetic tree construction using the neighbor-joining method and 1000 bootstrap replicates.

### Quantitative reverse transcription-PCR (qRT-PCR)

The RNA extraction, cDNA synthesis, qRT-PCR analysis, and relative quantification (containing three biological replicates) were described previously [8]. The amplification of *PsActin* and *NtUBC2* sequences were used as the internal control. The primers were listed in Table S2 (see online supplementary material). All analyses contained three technical replicates.

### Transient expression assays

The full-length *PsMYB10.1*, *PsbHLH3,* and *PsERF1B* coding regions from the flesh of the ‘Friar’ plum cultivar were inserted into the pSAK277 binary vector (with permission from Plant&amp; Food Research) under the control of the CaMV 35S promoter. The resulting binary vectors were then transformed into *Agrobacterium tumefaciens* strain GV3101 according to freeze–thaw method. After two-day incubation at 28°C, GV3101 was resuspended in infiltration buffer comprising 10 mmol L^−1^ MES monohydrate, 10 mmol L^−1^ MgCl_2_, and 150 mmol L^−1^ acetosyringone (pH 5.7) to an OD_600_ of 0.5 (Fang *et al.* [20]). After three hours of standing without light, separate strains were co-infiltrated into the back of leaves of *Nicotiana tabacum* with the help of needleless syringes. After incubation for 24 hours in the dark, the tobacco was transferred to regular conditions (16-hours light and 8-hours darkness) for incubation. Phenotypical changes in tobacco leaves were taken at the fifth day after treatment.

The fruitlets mature of ‘Friar’ plums were transiently transformed by the injection method. Agrobacterium mixture (0.2 mL) was injected into the equatorial part of the plum at a depth of 1 cm in the flesh. The agrobacterium mixture containing empty vector pSAK277 was injected into the symmetrical part of the equator as a control. The plum fruit were stored at room temperature for five days. Flesh tissues were taken from the area surrounding the infiltration site and used for the detection of the anthocyanin content and qRT-PCR analysis.

### VIGS assays

For RNAi-induced silencing of gene expression, a 291-bp *PsMYB10.1*-specific DNA fragment (bases 350–640 bp) located at the 3′ region of the cDNA was inserted into plasmids pTRV2. Recombinant vector and empty vectors were individually transformed into *A. tumefaciens* strain GV3101. GV3101 was incubated and resuspended to an OD_600_ of 0.5. The infestation solution containing pTRV1 was mixed fully with the infestation solution containing pTRV2 or pTRV2-*PsMYB10.1* in the ratio of 1:1. After three hours of standing without light, the mixed infestation solution was infiltrated into the plum flesh. The plums were stored at 23°C for six hours after removal from four-week storage at 0°C. Then, the plums were injected and incubated as described above.

### Yeast two-hybrid (Y2H) experiment

The full-length *PsMYB10.1* CDS was fused into the pGBKT7 vector, whereas the full-length *PsERF1B* CDS was fused into the pGADT7 vector. Four combinations were set up in the following way: pGBKT7-*PsMYB10.1* and pGADT7*-PsERF1B*, pGBKT7-*PsMYB10.1* and empty vector pGADT7, empty vector pGBKT7 and pGADT7*-PsERF1B*, empty vector pGBKT7 and pGADT7. The different recombinant plasmids were transformed into Y2H Gold yeast strain. Cells of the Y2HGold yeast strain harboring the recombinant plasmids were cultured on SD/−Leu –Trp medium initially, and then transferred to SD/−Leu –Trp –His –Ade medium. Because PsMYB10.1 had strongly self-activating activity, different concentrations of Aureobasidin A (AbA) were added to the SD/−Leu –Trp –His –Ade medium to suppress the background expression of PsMYB10.1. Eventually it was found that the growth of Y2HGold yeast carrying pGBKT7-*PsMYB10.1* and empty vector pGADT7 was completely inhibited when AbA was added at a concentration of 600 μg L^−1^. Based on the result of self-activation capacity assay, two groups of Y2HGold yeast strain containing pGBKT7-*PsMYB10.1* and pGADT7*-PsERF1B* or pGBKT7-*PsMYB10.1* and empty vector pGADT7 were dotted on the SD/−Leu –Trp –His –Ade containing 600 μg L^−1^ AbA medium and SD/−Leu –Trp –His –Ade containing AbA and X-α-gal medium to test for protein interactions.

### Luciferase complementation assay

The coding sequences of *PsMYB10.1* was inserted into the pCAMBIA1300-nLUC vector, whereas the coding sequences of *PsERF1B* was inserted into the pCAMBIA1300-cLUC vector. The resulting binary vectors were then transformed into *A. tumefaciens* strain GV3101 according to the freeze–thaw method. GV3101 was incubated and resuspended to an OD_600_ of 0.8. The agrobacterium infestation solution containing recombinant plasmid or empty vector mixed in the ratio of 1: 1. The mixed infestation solution containing GUS-nLUC and GUS-cLUC was used as positive control. After three hours of standing without light, the mixed infestation solution was infiltrated into the tobacco leaves with the help of needleless syringes. After 24 hours in the dark, the tobacco was transferred to normal conditions (16 hours of light and 8 hours of darkness) for 24 hours. Subsequently, the tobacco leaves were added with 1 mmol L^−1^ luciferin (Promega, Madison, WI, USA). The resulting luciferase signals were collected using the Tanon-5200 image system (Tanon Co., Shanghai, China).

### Dual-luciferase activity assay

The region ~2000 bp at the upstream of start codon ATG was regarded as the promoter of *PsUFGT* (proPsUFGT). The *cis*-acting elements of the promoter of *PsUFGT* were predicted by PlantCARE (http://bioinformatics.psb.ugent.be/webtools/plantcare/html/). The promoter of *PsUFGT* from the leaves of the ‘Friar’ plum cultivar was inserted into the pGreen II 800 binary vector upstream of the LUC gene, creating the reporter vector. The full-length *PsMYB10.1*, *PsbHLH3,* and *PsERF1B* coding regions were inserted into the pGreen II 62 SK binary vector, creating the effector vector. The recombinant vectors were then incorporated into *A. tumefaciens* EHA105 (pSoup) cells. After two-day incubation at 28°C, EHA105 (pSoup) was resuspended in infiltration buffer to an OD_600_ of 0.8. The *A. tumefaciens* strains carrying effector vectors (empty, *PsMYB10.1*, *PsbHLH3*, and *PsERF1B*) were mixed in different combinations equally and then combined with reporter vector in a 9:1 (v : v) ratio. After three hours of standing without light, the mixed infestation solution was infiltrated into the tobacco leaves [41]. After 24 hours in the dark, the tobacco was transferred to normal conditions for 24 hours. Dual-luciferase activity was measured using a Dual-Luciferase Reporter Assay System (Promega, Beijing, China). Three biological replicates were prepared for each sample.

### Statistical analysis

All experiments were performed at least including three replicates. Significance analysis of the data was conducted using SPSS 25.0 statistical software (IBM SPSS, Inc., Chicago, IL, USA). Statistical significance was computed using Tukey test or *t* test (*P* < 0.05).

## Acknowledgements

Thanks to professor Huazhong Ren, Xingwang Liu, Guiqin Qu and Benzhong Zhu (China Agricultural University) for providing experimental instruction and vectors.

Thanks to Dr Andrew Allan (The New Zealand Institute for Plant & Food Research Limited) and Dr Jun Wu (Nanjing Agricultural University) for kindly providing pSAK277 vector. Thanks to Dr Zhizhen Fang (Fujian Academy of Agricultural Sciences) for providing experimental instruction. This work was supported by the National Natural Science Foundation of China (No. 31872907 and No. 32272371), S & T Program of Hebei (No. C2021201011), and the 2115 Talent Development Program of China Agricultural University.

## Author contributions

R.X.: data curation, formal analysis, investigation, software, writing – original draft preparation. Y.W.: investigation. L.W.: writing – review and editing. Z.Z.: providing critical comments on manuscript editing. Funding acquisition. J.C.: conceptualization, writing – review and editing, supervision, project administration, funding acquisition. D.F.: methodology, supervision, investigation, data curation. W.J.: providing comments and helping to write the final manuscript.

## Data availability

All relevant data are included in the article and its supplementary file.

## Conflict of interest statement

The authors report no declarations of interest.

## Supplementary data

Supplementary data is available at *Horticulture Research* online.

## Supplementary Material

Web_Material_uhad091
