# Supplementary material for: PsERF1B-PsMYB10.1-PsbHLH3 module enhances anthocyanin biosynthesis in the flesh-reddening of amber-fleshed plum (cv. Friar) fruit in response to cold storage
Source: Hortic Res. 2023 May 4;10(6):uhad091. doi: 10.1093/hr/uhad091 (PMC10277908; doi:10.1093/hr/uhad091)
Supplement: Web_Material_uhad091 [file web_material_uhad091.zip › Supplementary table S1-2 and figure S1-2.docx]

Supplementary data

Table S1 TOP10 R2R3-MYB

| Name | GeneID | Length | log_2_FoldChange (ColdS4w/Harvest) | log_2_FoldChange (ColdS6w/Harvest) |
| --- | --- | --- | --- | --- |
| MYB10.1 | novel_G001502 | 720 | 8.214081839 | 10.35794238 |
| MYB108 | evm.TU.Chr3.41 | 897 | 7.349470301 | 7.246693598 |
| MYB6 | evm.TU.Chr1.420 | 951 | 6.566796499 | 6.951701837 |
| PHR1 | evm.TU.Chr1.5876 | 1518 | 5.907687681 | 6.778723388 |
| MYB4 | evm.TU.Chr1.2607 | 702 | 4.265433922 | 6.439117236 |
| PHR1 | evm.TU.Chr1.5810 | 936 | 5.458651435 | 6.271037855 |
| uncharacterized | evm.TU.Chr7.1059 | 2106 | 4.224357843 | 5.05048993 |
| PHL6 | evm.TU.Chr3.265 | 2049 | 3.252871315 | 3.895791209 |
| PHR1 | evm.TU.Chr6.3614 | 2298 | 3.300961919 | 3.773963405 |
| MYB308 | evm.TU.Chr2.1948 | 882 | 2.585655039 | 3.773494987 |

MYBs are sequenced based on the data of log_2_foldchange (ColdS4w/Harvest).

Table S2 Primer sequences used for quantitative reverse transcription-PCR

| Gene name | Gene ID | Functional annotation | Direction | Primer sequence (5’-3’) |
| --- | --- | --- | --- | --- |
| PsPAL | evm.TU.Chr6.2407 | phenylalanine ammonia-lyase | Forward  Reverse | AATGCAAGCACTTCAATCTTCC  GAACTCCAGCACTTCCACTATCC |
| PsCHS | evm.TU.Chr1.5839 | chalcone synthase | Forward  Reverse | AGAGTCCAAATTAGCCCTGAAGC  AATAAATAGCACACAGGCACTGG |
| PsCHI | evm.TU.Chr2.2237 | chalcone--flavonone isomerase | Forward  Reverse | TAAGTTCACGGCGATTGG  CGGCTTCTGGTATGGATG |
| PsC4H | evm.TU.Chr6.365 | trans-Cinnamate 4-monooxygenase | Forward  Reverse | GTCATCGTCGCAATCACT  CTTCTTCACATCCTCAACCA |
| PsF3H | evm.TU.Chr2.2487 | flavanone 3-hydroxylase | Forward  Reverse | AGGTGCCATTCTTGACTG  CTGTGCTCTACGCTCTTAT |
| PsF3’H | evm.TU.Chr5.440 | flavonoid 3'-hydroxylase | Forward  Reverse | CCACGACACCAACTTCTC  TCTCCACAACCATCTCCTT |
| PsDFR | evm.TU.Chr1.2057 | dihydroflavonol-4-reductase | Forward  Reverse | AGCAGGAACCGTGAATATC  AGAGGTCGTCCAAGTGAA |
| PsANS | evm.TU.UTG5995.3 | anthocyanidin reductase | Forward  Reverse | GACTACTTCTTCCACCTTGT  ATCCTCACCTTCTCCTTGT |
| PsUFGT | evm.TU.Chr2.3164 | UDP-glucose flavonoid 3-Oglucosyltransferase | Forward  Reverse | ACAATCCAACAACTCACTCT  GAAGGCGTCGGTAATCAA |
| PsLAR | evm.TU.Chr1.2785 | leucoanthocyanidin reductase | Forward  Reverse | CGGAGGAGCCAGGGCTGAGT  GGGAGAACATCGGCGGGGTG |
| PsGST | evm.TU.Chr3.135 | glutathione S-transferase | Forward  Reverse | CAAGTTCCAGTAGTAGAAGATG  CCACCAAGCATTCACATTC |
| PsbHLH3 | evm.TU.Chr8.2504 |  | Forward  Reverse | ACCATCACCGACACCTAT  TTCACATTCTCCTTCACCTT |
| PsMYB10.1 | novel_G001502 |  | Forward  Reverse | CGGAAGATGAAGTAGAT  TGTTGATGGTGATGATTGTG |
| PsERF1B | evm.TU.Chr8.2346 |  | Forward  Reverse | GATGCTGCTCTATGATGCT  TGGAGTGCTTCCTCTTGA |
| PsActin | evm.TU.Chr6.1716 |  | Forward | CTGGACCTTGCTGGTCGT |
|  |  |  | Reverse | ATTTCCCGCTCAGCAGTG |
| NtF3’H | AB289449.1 | flavonoid 3'-hydroxylase | Forward  Reverse | GTCCTCAAGCACAGTAGAA  TTCCTCGCACATCAACTT |
| NtANS | AB289447.1 | anthocyanidin reductase | Forward  Reverse | TTAACTACTACCCCAAATGTCC  TGCCGTTACCCACTGTCCTTC |
| NtDFR | AB289448.1 | dihydroflavonol-4-reductase | Forward  Reverse | CAGAGAAGGCCGCAATGGAAGC  GGTGGGAATGTAGGCGTGAGGAAT |
| NtUFGT | FG627024.1 | UDP-glucose flavonoid 3-Oglucosyltransferase | Forward  Reverse | CAATGAGTGCATTGGATGCC  CCAGCTCCATTAGGTCCTTG |
| NtUBC2 | AB026056 |  | Forward  Reverse | TGAGAACAAGCGCGAATACAACAG  AACAGATTAAGAGTGCGGGAGATG |


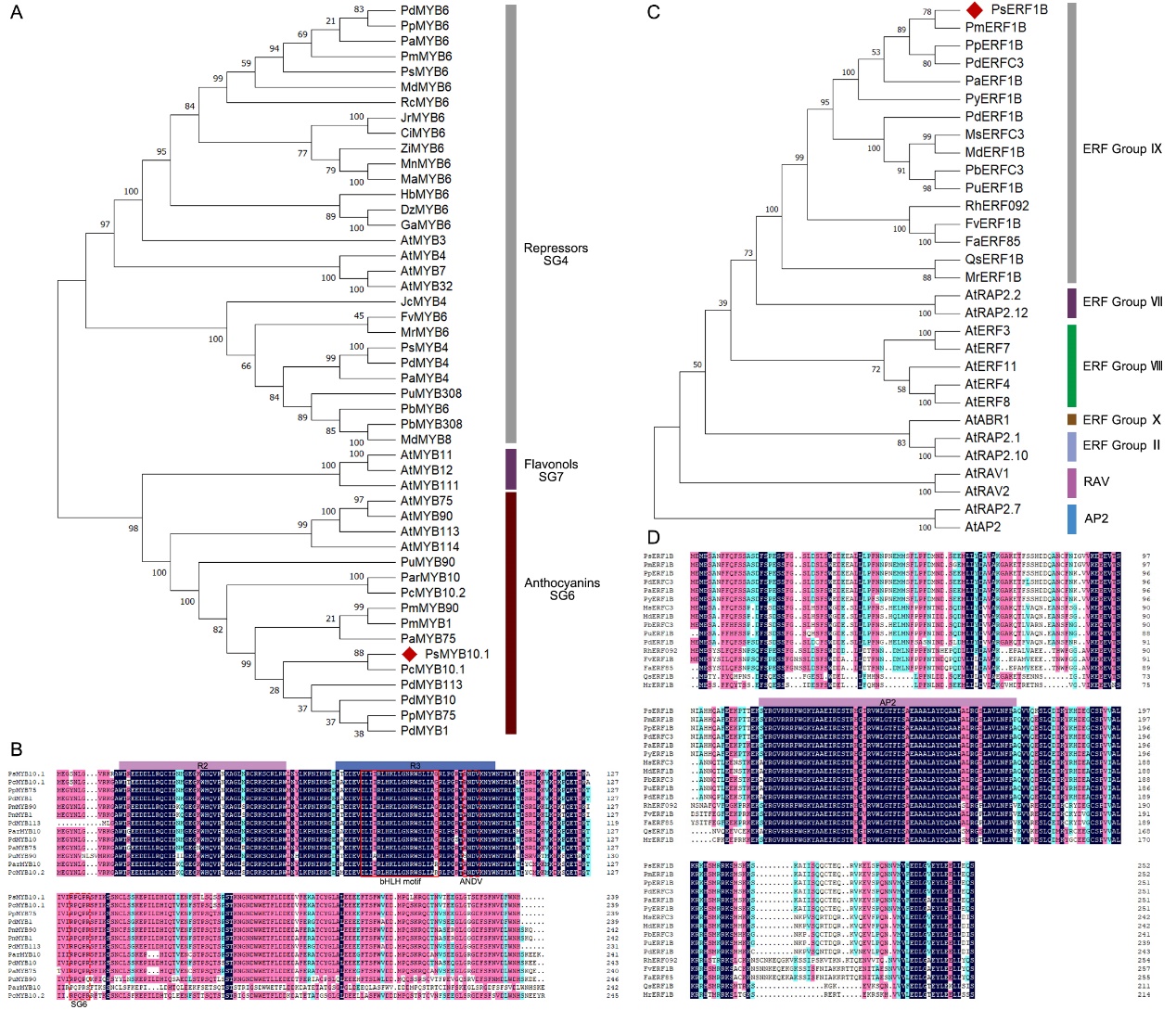


Fig S1 Phylogenetic tree analysis and protein sequence alignment of PsMYB10.1 and PsERF1B. A, phylogenetic tree analysis of PsMYB10.1 protein and related MYB TFs from diﬀerent species. B, protein sequence alignment of PsMYB10.1. C, phylogenetic tree analysis of PsERF1B protein and related TFs from diﬀerent species. D, protein sequence alignment of PsERF1B. NCBI accession numbers of MYB TFs are as follows: PdMYB6 (XP_034208131.1), PpMYB6 (XP_007222954.1), PaMYB6 (XP_021801177.1), PmMYB6 (XP_008219033.1), MdMYB6 (XP_028950860.1), RcMYB6 (XP_024173701.1), JrMYB6 (XP_018841121.2), CiMYB6 (XP_042969580.1), ZiMYB6 (XP_015886342.1), MnMYB6 (XP_010104477.1), MaMYB6 (ATL77060.1), HbMYB6 (XP_021662613.1C), DzMYB6 (XP_022773528.1), GaMYB6 (XP_017616416.1), AtMYB3 (AT1G22640), AtMYB4 (AT4G38620), AtMYB7 (AT2G16720), AtMYB32 (AT4G34990), JcMYB4 (XP_012072198.1), FvMYB6 (XP_004297358.1), MrMYB6 (KAB1209240.1), PdMYB4 (BBG94536.1), PaMYB4 (XP_021824312.1), PuMYB308 (KAB2621156.1), PbMYB6 (XP_018502946.1), PbMYB308 (XP_009357784.1), MdMYB8 (XP_008352913.1), AtMYB11 (AT3G62610), AtMYB12 (AT2G47460), AtMYB111 (AT5G49330), AtMYB75 (AT1G56650), AtMYB90 (AT1G66390), AtMYB113 (AT1G66370), AtMYB114 (AT1G66380), PuMYB90 (KAB2612362.1), ParMYB10 (ABX71490.1), PcMYB10.1 (AKV89248.1), PmMYB90 (XP_008244325.1), PmMYB1 (AYV88909.1), PaMYB75 (XP_021811998.1), PcMYB10.1 (AKV89247.1), PdMYB113 (BBG99695.1), PdMYB10 (ABX71491.1), PpMYB75 (XP_007216530.1), PdMYB1 (XP_034208044.1). NCBI accession numbers of AP2/EREBP TFs are as follows: PmERF1B (XP_008235690.1), PpERF1B (XP_007200770.2), PdERFC3 (XP_034228340.1), PaERF1B (XP_021814829.1), PyERF1B (PQQ17832.1), MsERFC3 (XP_050107909.1), MdERF1B (XP_008349063.1), PbERFC3 (XP_009369492.2), PuERF1B (KAB2602276.1), RhERF92(AYW51734.1), FvERF1B (XP_004289932.1), FaERF85 (AZL19487.1), QsERF1B (XP_023906372.1), MrERF1B (KAB1214394.1), AtRAP2.2 (AT3G14230), AtRAP2.12 (At1G53910), AtERF3 (NM_103946.3), AtERF7 (NM_112922.3), AtERF3 (NM_103946.3), AtERF7 (NM_112922.3), AtERF11 (NM_102603.3), AtERF4 (NM_112384.2), AtERF8 (NM_104196.3), AtABR1 (AT5G64750), AtRAP2.1 (BT024691.1), AtRAP2.10 (AT4G36900), AtRAV1 (AB013886.1), AtRAV2 (AB013887.1), AtRAP2.7 (NM_001202696.2), AtAP2 (NM_119856.3).


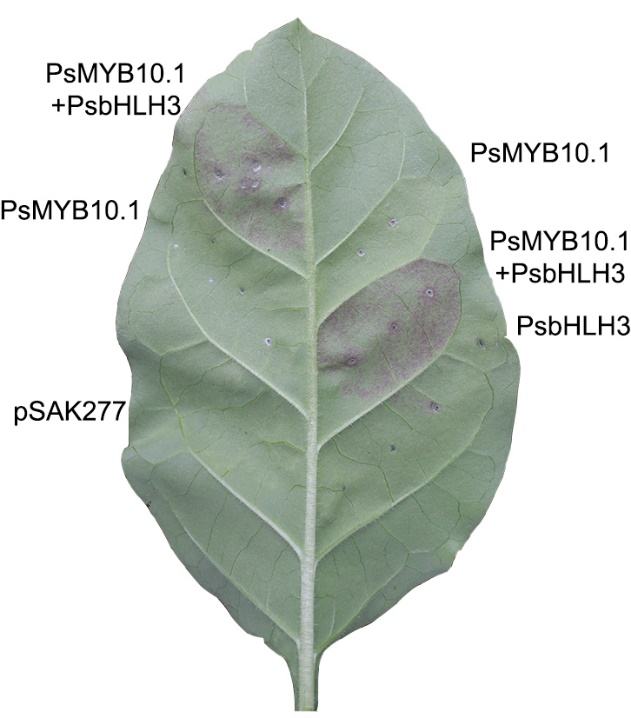


Fig S2 Transient expression assays in tobacco leaves
